# Comparative Analyses of Homocitrate Synthase Genes of Ascomycetous Yeasts

**DOI:** 10.1155/2012/254941

**Published:** 2012-03-18

**Authors:** Hiromi Nishida

**Affiliations:** Agricultural Bioinformatics Research Unit, Graduate School of Agricultural and Life Sciences, University of Tokyo, Tokyo 113-8657, Japan

## Abstract

Most ascomycetous yeasts have 2 homocitrate synthases (HCSs). Among the fungal lysine biosynthesis-related genes, only the HCS gene was duplicated in the course of evolution. It was recently reported that HCS of *Saccharomyces cerevisiae* has an additional function in nuclear activities involving chromatin regulation related to DNA damage repair, which is not related to lysine biosynthesis. Thus, it is possible that the bifunctionality is associated with HCS gene duplication. Phylogenetic analysis showed that duplication has occurred multiple times during evolution of the ascomycetous yeasts. It is likely that the HCS gene duplication in *S. cerevisiae* occurred in the course of *Saccharomyces* evolution. Although the nucleosome position profiles of the two *S. cerevisiae* HCS genes were similar in the coding regions, they were different in the promoter regions, suggesting that they are subject to different regulatory controls. *S. cerevisiae* has maintained HCS activity for lysine biosynthesis and has obtained bifunctionality.

## 1. Introduction

Organisms synthesize lysine from 2-oxoglutarate through *α*-aminoadipate or from aspartic acid through diaminopimelate [1]. Animals cannot synthesize lysine. Fungi synthesize lysine through *α*-aminoadipate [2–4]. The other eukaryotes synthesize lysine through diaminopimelate. Archaea and bacteria were also believed to synthesize lysine through diaminopimelate until it was reported that the extremely thermophilic bacterium *Thermus thermophilus* synthesizes lysine through *α*-aminoadipate [5–8].

During lysine biosynthesis in the budding yeast *Saccharomyces cerevisiae*, *α*-aminoadipate is synthesized from 2-oxoglutarate and acetyl-CoA by the enzymes Lys20 or Lys21 (homocitrate synthase [HCS]), Lys4 (homoaconitase), Lys12 (homoisocitrate dehydrogenase), and *α*-aminoadipate aminotransferase [9]. Lysine is synthesized from *α*-aminoadipate by the enzymes Lys2 (aminoadipate reductase), Lys5 (phosphopantetheinyl transferase which posttranslationally modifies Lys2), Lys9 (saccharopine dehydrogenase, glutamate forming), and Lys1 (saccharopine dehydrogenase, lysine forming) [1, 4].

It has been unclear why *S. cerevisiae* has 2 HCSs (Lys20 and Lys21). For example, homocitrate is mainly synthesized through Lys21 during growth on ethanol, while under fermentative metabolism, Lys20 and Lys21 play redundant roles [11]. It was recently reported that Lys20 of *S. cerevisiae* functions in nuclear activities involving chromatin regulation that are distinct from its previously established role in lysine synthesis [12]. Lys20 of *S. cerevisiae* is linked to the DNA damage repair process via the histone acetyltransferase Esa1 and the H2A.Z histone variant [12]. Thus, it is possible that this bifunctionality is associated with HCS gene duplication.

## 2. Materials and Methods

### 2.1. Phylogenetic Analyses

I selected 71 HCSs (31 from Saccharomycotina species, 30 from Pezizomycotina species, 2 from Taphrinomycotina species, and 8 from Basidiomycota species) based on BLASTP results in the fungal genome database at NCBI (http://www.ncbi.nlm.nih.gov/projects/genome/guide/fungi/). Multiple alignments were generated with CLUSTAL W. A maximum likelihood tree was reconstructed using MEGA version 5 [10]. The WAG model was used as the amino acid substitution model. The nearest neighbor interchange was used as the maximum likelihood heuristic method. The *γ*-distributed rate was considered, and the number of discrete gamma categories was 3.

### 2.2. Nucleosome Position Comparison

Nucleosome positioning was used to compare gene promoter regions. I used nucleosome position data from *S. cerevisiae* BY4741 [13]. The nucleosome position profiles were compared between the promoter (1000 bases upstream of the translational start site) and coding regions (between the translational start and end site) of the HCS genes, according to a previously described method [14]. Similarity between the two nucleosome position profiles was estimated using the Spearman's rank correlation coefficient.

## 3. Results and Discussion

**Figure 1:**
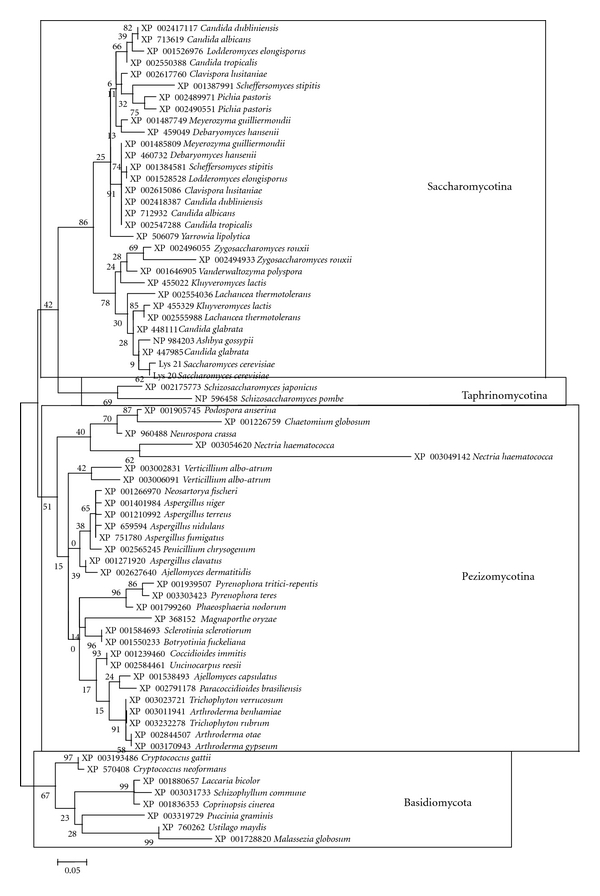
Phylogenetic relationships among 71 fungal homocitrate synthases. The phylogenetic tree was constructed based on multiple alignment with complete deletion of gap sites using the maximum likelihood method of MEGA software [10] with 100 bootstrap analyses. The WAG model was used as the amino acid substitution model. A total of 103 amino acid sites were considered. The *γ*-distributed rate was considered, and the number of discrete gamma categories was 3. The gamma was 0.81; the discrete rates were 0.14, 0.65, and 2.2.

The HCS phylogenetic tree (Figure 1) indicates that the HCS gene has been duplicated multiple times in the course of ascomycete evolution. The 31 HCSs of the Saccharomycotina species (ascomycetous yeasts) are encoded in 17 organisms. In contrast, the 30 HCSs of the Pezizomycotina species (filamentous ascomycetes) are encoded in 28 organisms. Thus, 14 of the 17 Saccharomycotina species and 2 of the 28 Pezizomycotina species have 2 HCSs (Figure 1).

Gene duplication is not found in *LYS1*, *LYS2*, *LYS5*, *LYS9*, and their homologues [15]. In addition, no duplication was found in *LYS4*, *LYS12*, and their homologues (data not shown). Therefore, among the fungal lysine biosynthesis-related genes, only the HCS gene has been duplicated. Phylogenetic analysis of HCSs in ascomycetous yeasts showed that the *S. cerevisiae* HCSs (Lys20 and Lys21) are most closely related to each other (Figure 1), suggesting that HCS gene duplication occurred during evolution of the genus *Saccharomyces*. On the other hand, all Saccharomycotina species except *Ashbya gossypii*, *Vanderwaltozyma polyspora*, and *Yarrowia lipolytica* have duplicated HCS genes (Figure 1). Thus, HCS gene duplication may be related to genome duplication events in Saccharomycotina [16–18].

**Figure 2:**
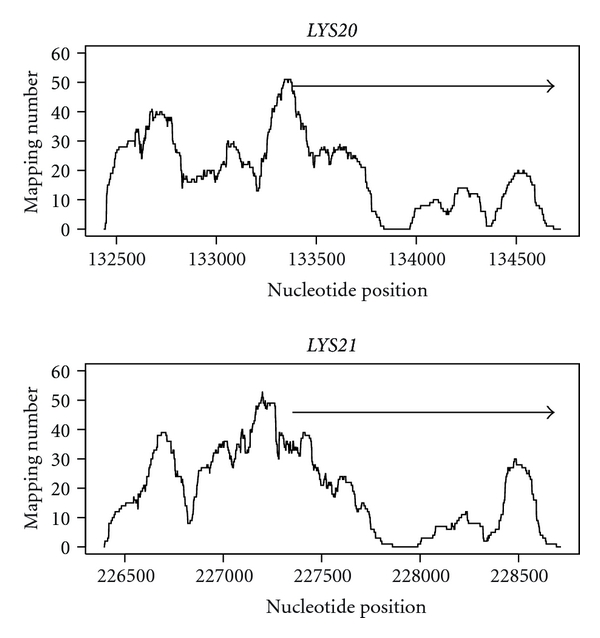
Mapping of nucleosomes around *Saccharomyces cerevisiae LYS20* and *LYS21*. In this study, I used nucleosome position data from *S. cerevisiae* BY4741 [13]. Based on each nucleosomal DNA fragment sequence, nucleosomal mapping numbers were estimated for each nucleotide position [14]. Arrows indicate the coding region.

In addition to the phylogenetic analysis based on HCS amino acid sequences, I compared the nucleosome positioning of *LYS20* and *LYS21*. Interestingly, nucleosomes were mapped to the HCS gene promoters more often than to the coding regions (Figure 2). Nucleosome position profiles in the coding regions were highly correlated (Spearman's rank correlation coefficient = 0.833) between *LYS20* and *LYS21*. On the other hand, those in the gene promoter regions were poorly correlated (Spearman's rank correlation coefficient = 0.396). This result suggests that these 2 HCS genes have different regulatory systems.

On the other hand, *LYS20* expression is most similar to *LYS21* expression, and *LYS21* is most similar to *LYS20* expression, based on the SPELL version 2.0.2 [19]. In addition, recent comparative analyses of orthologous genes in evolutionarily close yeasts indicated that divergence of nucleosome positioning is not correlated with divergence of gene expression [20, 21].

Although HCS (Lys20 and Lys21) is located in the nucleus of *S. cerevisiae* [22], HCS is located in the cytoplasm of *Penicillium chrysogenum* [23, 24]. *P. chrysogenum* has a single HCS gene (Figure 1). The phylogenetic tree (Figure 1) showed that gene duplication is not found in Basidiomycota and Taphrinomycotina. In addition, gene duplication has occurred rarely in Pezizomycotina, suggesting that a common ancestor of the Dikarya lacked the nuclear function of chromatin regulation. Considering that duplication of the HCS gene occurred in a limited number of ascomycetes, it may not be an essential event in the evolution of Dikarya. I hypothesize that after divergence of the phyla Ascomycota and Basidiomycota, *S. cerevisiae* obtained HCS bifunctionality.
